# Rota-Lithotripsy as a Novel Bail-Out Strategy for Highly Calcified Coronary Lesions in Acute Coronary Syndrome

**DOI:** 10.3390/biomedicines10112795

**Published:** 2022-11-03

**Authors:** Piotr Rola, Łukasz Furtan, Szymon Włodarczak, Jan Jakub Kulczycki, Mateusz Barycki, Marek Szudrowicz, Michalina Kędzierska, Anna Pszonka, Justyna Korus, Adrian Doroszko, Maciej Lesiak, Adrian Włodarczak

**Affiliations:** 1 Faculty of Health Science and Physical Culture, Witelon Collegium State University, 59-220 Legnica, Poland; 2 Department of Cardiology, Provincial Specialized Hospital, 59-220 Legnica, Poland; 3 Department of Cardiology, The Copper Health Centre (MCZ), 59-300 Lubin, Poland; 4 Faculty of Medicine, Wroclaw Medical University, 50-556 Wroclaw, Poland; 5 Clinical Department of Internal Medicine and Occupational Diseases, Hypertension and Clinical Oncology, Faculty of Medicine, Wroclaw Medical University, 50-556 Wroclaw, Poland; 6 1st Department of Cardiology, University of Medical Sciences, 61-484 Poznan, Poland

**Keywords:** rota-lithotripsy, rota-tripsy, rotational atherectomy, intravascular lithotripsy, shockwave device, calcified lesions, lesion preparation, novel therapeutic option, clinical outcome, acute coronary syndrome (ACS)

## Abstract

Background: Heavily calcified lesions in acute coronary syndrome (ACS) still represent a challenging subset for percutaneous coronary intervention (PCI). Rota-lithotripsy—a marriage of rotational atherectomy and intravascular lithotripsy—has recently been introduced to clinical practice as a novel therapeutic option. Methods: This study is among the to present the 6-month clinical outcomes of rota-lithotripsy when performed in the ACS setting. The study cohort consisted of 15 consecutive ACS patients who underwent a rota-lithotripsy-PCI due to the presence of a highly calcified, undilatable lesion. Results: The procedural success ratio reached 100%. During the 6-month follow-up, in two of the patients, instances of MACE (major adverse cardiac events) occurred, including one fatal event. Additionally, during the observation period, one target lesion failure, due to subacute stent thrombosis, was identified. Conclusions: Rotational atherectomy with the subsequent use of shockwave intravascular lithotripsy appears to be a safe and effective therapeutic bail-out option for the management of highly calcified coronary artery lesions. Despite, these initial favorable outcomes, carrying out a large number of studies with long-term observations is still necessary in order to establish the potential benefits and shortcomings of rota-lithotripsy.

## 1. Introduction

Calcifications in the coronary vessels imply the presence of coronary artery disease (CAD) and are a well-established risk factor for unfavorable clinical events [1]. Despite the ongoing development of percutaneous coronary intervention (PCI) techniques and remarkedly improved armamentarium outcomes, highly calcified coronary lesions remain a challenge and strongly determine the results of percutaneous surgery. High calcium burden increases the procedural complexity affecting the lesion preparation, stent delivery, and adequate stent implantation, which subsequently results in a higher rate of periprocedural complications along with suboptimal long-term clinical outcomes. In particular, an appropriate lesion preparation appears to be a crucial point during the PCI of highly calcified coronary lesions [2]. Currently, numerous calcium modification devices are available and have recently become part of contemporary practice [3]. The variety of PCI-related devices—including dedicated balloon-dependent catheters (non-compliant, OPN, cutting, and scoring balloons), atherectomy devices (rotational, orbital, or laser) [4], as well as novel intravascular lithotripsy technology [5,6]—has allowed for the predictable and safe treatment of patients with calcified coronary lesions. The usefulness of each device depends on the nature of the calcium burden and clinical context. In some specific, highly demanding subpopulations, the utilization of several different devices is necessary in order to achieve a crack in the calcium deposits. It must be noted that a step-wise progression should be considered once the standard maneuvers are unsuccessful. However, such an approach may increase the procedural complexity resulting in a higher complication rate, including the possibility of an acute coronary syndrome (ACS) subset arising.

Rota-lithotripsy, also known as rota-tripsy, is a combination of rotational atherectomy with subsequent intravascular lithotripsy [7,8,9,10]. Further, it has recently been introduced to clinical practice and is applied as a bail-out strategy for undilatable calcified coronary lesions. Nevertheless, there are scarce data regarding this novel strategy in the ACS setting; in addition, the current literature is limited to several case report studies [11,12,13,14,15,16].

## 2. Materials and Methods

### 2.1. Study Population

An investigation into a total of 15 consecutive ACS patients who underwent a series of PCI treatments (with the support of rotational atherectomy, as well as with the subsequent bail-out use of intravascular lithotripsy due to the presence of an undilatable lesion) was conducted. The procedures were performed at two cooperative high-volume (at least 1000 PCI procedures annually) cardiology departments in the Lower Silesia Region in Poland. All the PCI procedures were performed from May 2019 to February 2022. During this period, in both cardiac centers, no other alternative methods (including the burr size escalation maneuver, etc.) of management with undilatable lesions had been used.

**Figure 1:**
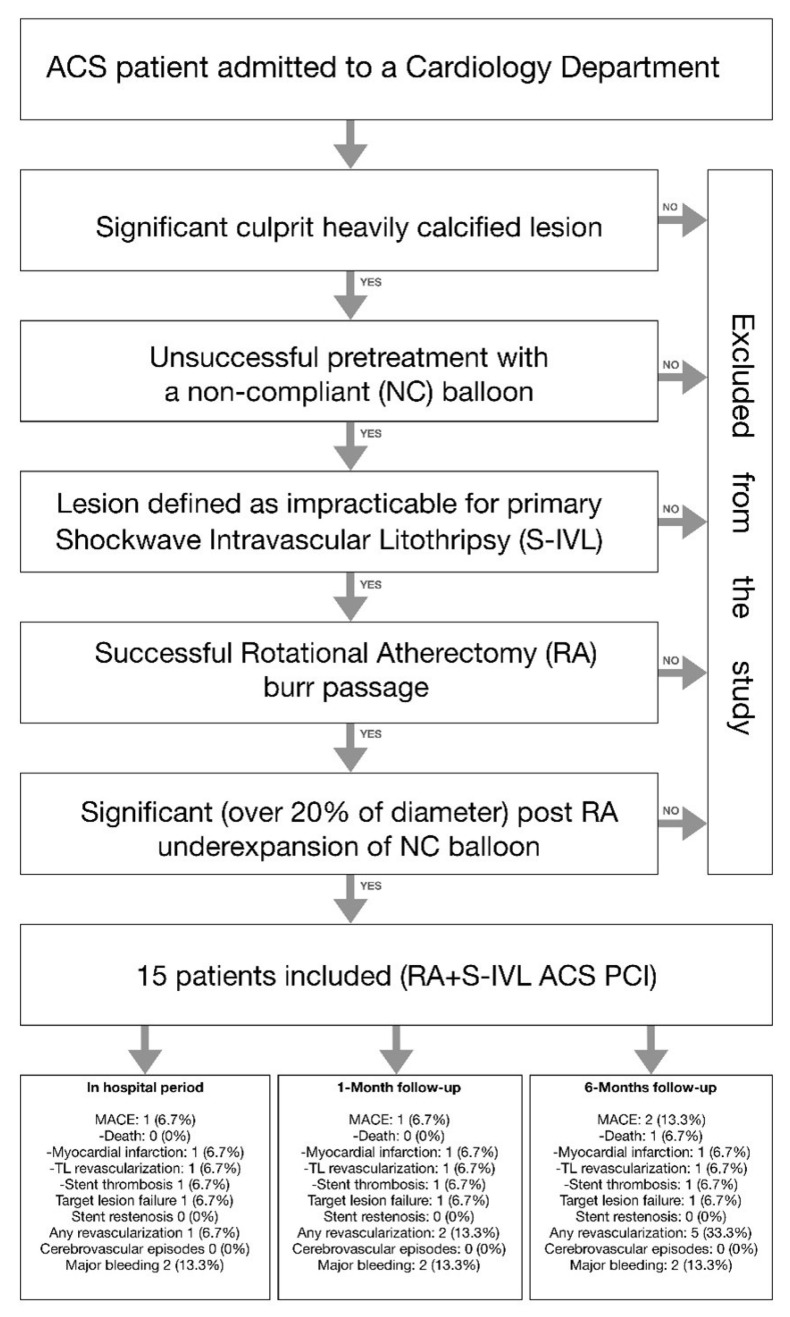
Study inclusion and exclusion criteria.

The main inclusion criteria were the presence of a culprit calcified lesion treated with a rotational atherectomy (RA) device due to initially unsuccessful pretreatment with a non-compliant (NC) balloon. Additionally, the lesion had to be defined by the operator as impracticable for primary S-IVL (mainly long lesions with high-grade stenosis). In all involved cases—despite the successful passage of a rotational atherectomy burr still—a significant (over 20% in diameter) under-expansion of the NC balloon (sized 1:1 to vessel references) was observed. A flow chart of the study design is presented in Figure 1.

### 2.2. PCI Procedures

The decision to perform the PCI was based either on the cardiology team’s judgment or on clinical indication (e.g., ongoing ischemia, patients’ lack of will, surgical treatment, etc.). All patients were thoroughly informed regarding the available therapeutic options and the PCI-related risks before written informed consent was provided. There were no vessel-related exclusion criteria regarding lesion anatomy, length, tortuosity, severity, or prior stent placement. All the clinical features regarding the PCI procedure including vascular access point; guiding catheter size; intravascular imaging guidance (OCT/IVUS); burr or shockwave balloon size (operators were encouraged to reach a burr/vessel ratio of 0.5 and S-IVL balloon/vessel ratio of 1.0); rotablation speed; number of ultrasonic pulses applied; and periprocedural pharmacotherapy, along with stenting technique, were left to the operators’ discretion.

### 2.3. Study Endpoints

The primary endpoint of the study was in the investigation of occurrences of major adverse cardiac events (MACE) during the hospitalization period, as well as during the 1- and the 6-month post-discharge observation periods. MACE was defined as: death, myocardial infarction, need for second target vessel revascularization, and probable or diagnosed in-stent thrombosis. The secondary endpoints included cerebrovascular episodes, target lesion failure, all kinds of revascularization procedures, major bleeding, and scaffold restenosis. The myocardial infarction definition was based on the fourth universal definition of myocardial infarction [17]. In addition, target lesion failure, any other revascularization, in-stent thrombosis, and stent restenosis were defined in accordance with the Academic Research Consortium-2 consensus document guidelines [18]. Major bleeding was defined as types 3 and 5, followed by the consensus that was achieved by the Bleeding Academic Research Consortium [19].

### 2.4. Statistical Analysis

The R language was used for analyses. Continuous variables were characterized by their mean and standard deviation. They were also characterized by median, and first and third quartiles (dependent on their distribution), whereas the frequencies were used for the purposes of categorical variables. The Shapiro–Wilk test was used to verify the normality of continuous variables. Further, the significance level was set to <0.05.

## 3. Results

The study population consisted of 15 patients, mainly males (86.7%), with a mean age of 70.9 ± 9.1 years. Most of the subjects had suffered from NSTEMI (73.4%) and were characterized by a high prevalence of cardiovascular risk factors such as hyperlipidemia (100%), hypertension (86.6%), diabetes (66.6%), and a previous history of MI (53.3%). All basal clinical data regarding the study cohort are pooled in Table 1.

**Figure 2:**
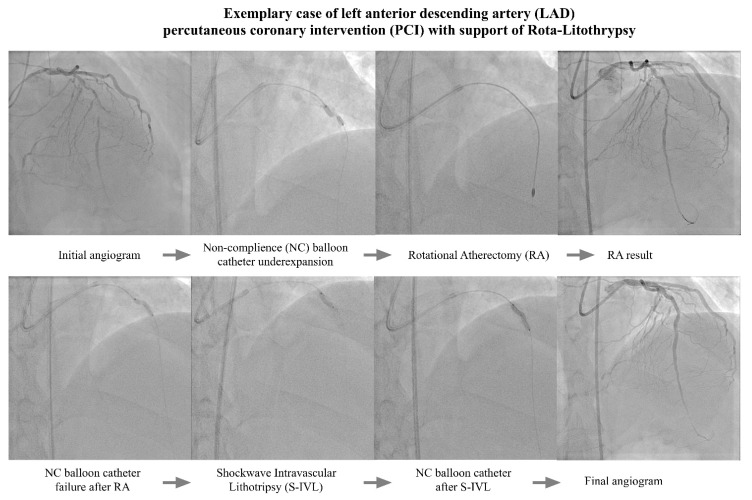
An exemplary PCI procedure.

Despite the initial high advancement of coronary artery disease (the mean Syntax I score reached 21.1 ± 10.9) and mild impairment of the left ventricular ejection fraction (44.8 ± 16.2), radial access was used in 80.0% of cases. However, most of the procedures were performed using a 7F (73.3%) catheter. The data regarding periprocedural features are presented in Table 2. The mean burr size was 1.55 ± 0.15 mm, and none of the subjects required enhancing of the burr size. The average rotational speed was set at 162,300 ± 4242 rpm, with a mean rotablation duration time of 111.7 ± 61.2 s. The subsequent intravascular lithotripsy was performed as a bail-out strategy, mainly due to the under-expansion of the non-compliant balloon catheter (sized angiographically, i.e., with a balloon/vessel ratio 1:1), after the passage of the rota burr through the lesion. A similar sizing rule was applied to the RA of the S-IVL balloon (3.2 ± 0.15 mm), and an average of 45.3 ± 19.9 pulses was used per procedure. The total DES length per procedure was relatively high (64 ± 29.7 mm), while the DES diameter exceeded 3 mm (3.16 ± 0.48 mm). Figure 2 presents a representative PCI procedure.

During the hospitalization period, one MACE related to a subacute stent (5 days after initial PCI) thrombosis of the target lesion was observed. Additionally, one patient underwent a scheduled PCI for a non-culprit lesion. In the study cohort, we observed two major occurrences of bleeding: one was related to the femoral access site (the patient required a blood transfusion and urgent surgery); the second was not directly related to the procedure (i.e., it was a periprosthetic leak from a previous (6 years before index PCI) implanted stent-graft to the abdominal aorta). There were no additional MACEs in the 30-day follow-up period. In one subject, a scheduled PCI of another vessel was performed. During the 6-month follow-up period, an additional episode of MACE was observed. Approximately 5 months after discharge, one death occurred. A patient with a high number of comorbidities and a low left ventricular ejection fraction (LVEF) (15–20%) with a previously implanted cardioverter–defibrillator (ICD) and newly diagnosed COVID-19 was admitted to the emergency department and died a few hours later with symptoms of acute heart failure. All data regarding clinical outcomes were collected in Table 3.

## 4. Discussion

Coronary artery lesions with a high calcium burden still represent a challenging task for interventional cardiologists. Further, these lesions are connected with a greater risk of periprocedural complications, as well as with a late failure due to the stent underexpansion and malapposition, consequently resulting in poor clinical outcomes [20,21,22]. Heavily calcified coronary plaques, particularly those with deep calcium deposits, are commonly resistant to the standard plaque modification techniques, including the conventional balloon angioplasty. This is of particular note due to the fact that profound calcium deposits are prone to be underestimated during classical coronary angiography (CA) [23], especially in the urgent subset of acute coronary syndromes (ACS). As a result, ad hoc decisions regarding percutaneous revascularization are made during the coronary angiography, with no time left for well-balanced planning. As a consequence, this may unacceptably increase the rate of periprocedural complications. Additionally, the presence of highly thrombotic “vulnerable plaques” in ACS patients is associated with a higher overall mortality and adverse cardiovascular events [24,25].

To tackle these highly demanding lesions, several debulking modalities (such as rotational, orbital, and laser atherectomy, or the recently introduced shockwave intravascular lithotripsy) have become a part of contemporary clinical practice. Despite the well-established safety and efficiency of the atherectomy devices, utilization in the ACS subset is burdened with worse outcomes compared to stable patients [26,27]. Due to the novelty of the shockwave device, the evidence supporting the use of S-IVL in the ACS subset comes mainly from case reports, studies or low-number registries, which is encouraging [28,29,30,31,32]. However, in some rare cases, the combination of the use of debulk devices and non-compliant balloon catheter lesion preparations is insufficient in order to achieve an adequate stent expansion.

Different mechanisms may be responsible for suboptimal lesion preparation in the context of calcified lesions. Atherectomy devices are mainly dedicated to long, tight lesions modifying calcium plaques, due to their high-speed (from 140,000 to 180,000 rpm in the case of rotational devices, and from 80,000 to 120,000 rpm in the case of orbital devices) rotating burrs, which perform atheroablation leading to the pulverization of calcified deposits. For that reason, atherectomy devices’ field of action is mainly limited to superficial plaques with a lack of impact on profound calcium deposits [33,34]. On the other hand, the NC balloon dilatation, in terms of eccentric calcium plaque, may direct the dilatation forces toward the non-calcified segments of the artery with a faint effect on calcium nodules. On the other hand, the S-IVL is a bulky device focused on the disruption of deep calcium plaque with the inability to cross through high-grade stenosis lesions [35,36,37].

In response to the shortcomings of listed plaque-modification methods, a novel technique of highly calcified lesion preparation rota-lithotripsy, also known as rota-tripsy or rota-shock, has been recently introduced. The initial low-number and short-term [2,9,14,27,28,38] observation studies point at promising results and an acceptable safety profile. This novel treatment concept is dedicated to highly calcified lesions, concentrically protruding to the vessel lumen and extending deep into the vascular wall. This specific plaque architecture limits the efficacy of classical single-device strategies involving either RA or -S-IVL in terms of lesion preparation before stenting. In fact, in this high-grade lesion, despite RA, NC balloons do not fully expand, thereby providing dog-bone effects and implying the need to use S-IVL in order to fracture deep deposits of calcium.

Even though the need for additional S-IVL may be reduced by an escalation of burr size, when the burr-to-artery ratio exceeds over 0.5/0.6, this leads to an increased rate of complications (i.e., slow flow phenomena, dissection, or perforations) [39,40,41,42]. In addition, this often implies the need for guiding size escalation or even force switching to the transfemoral approach, which additionally affects the amount of peri-procedural access-side bleeding [43,44]. Until rota-lithotripsy’s introduction to clinical practice, the burr escalation maneuver was considered a basic bailout strategy for primarily undilatable lesions. However, in an ACS subset with a high initial thrombogenic potential, the risk of periprocedural complications, particularly in regard to slow flow phenomena, was high [39,45].

To the best of our knowledge, this is the first description of a mid-term follow-up on the outcomes of the simultaneous use of rotational atherectomy and shockwave intravascular lithotripsy in terms of ACS subjects. The results obtained in our cohort indicate the safety and efficacy of the rota-lithotripsy treatment to high-risk subjects where the single-burr RA strategy is revealed to be insufficient for adequate lesion preparation.

A relatively high Syntax Score I (21.1 ± 10.9) along with a high Syntax II PCI score (39.0 ± 14.9) confirm the complexity and advancement of CAD in the study group. Despite an unfavorable clinical subset—i.e., in the ACS setting, the high lesion complexity as well as the considerable comorbidities observed in the study group—the mid-term MACE rate was comparable to the one observed in the ACS cohorts undergoing only rotational [46] or orbital atherectomy [47] without the subsequent use of an additional debulk device. Despite the fact that most of the procedures were performed via radial access (with the support of a 7F guiding catheter), approximately one out of seven patients expired with major bleeding. What must be emphasized, however, is that only one patient possessed access-site-related bleeding. Such a significant reduction in bleeding was possible due to the synergistic use of various calcium crack methods. The rota-lithotripsy technique allowed the practitioners to reduce the burr size to approximately 1.5 m, while most of the RA-related procedures were performed in an up-to-date manner, using a burr size of less than 1.75 mm [40,43].

Although only one non-fatal subacute stent thrombosis occurred in our study cohort, and we did not observe any other rota-lithotripsy-related periprocedural complications, further studies are required in order to address the safety concerns. The use of RA increases the rate of thrombotic events, particularly slow flow phenomena [45], and some concerns raised recently regarding a higher tendency toward platelet aggregation following the implementation of shockwave therapy [35]. As result, future studies are necessary in order to evaluate these potential disadvantages in the subsequent use of RA and S-IVL.

### Limitations

This is a non-randomized observational pilot study with a relatively small number of participants. Nevertheless, the procedures have scarcely been introduced in clinical practice and have still managed to obtain bail-out life-saving status in extremely high-risk patients. Moreover, for the same reason, the rate of intravascular guidance has been comparatively low [48].

## 5. Conclusions

To the best of our knowledge, this is the first description of mid-term follow-up outcomes of the simultaneous use of rotational atherectomy and shockwave intravascular lithotripsy in ACS subjects. Rotational atherectomy with the subsequent use of shockwave intravascular lithotripsy appears to be a safe and effective therapeutic bail-out option for the management of highly calcified coronary artery lesions. Further studies with a higher number of participants, a longer observation period, and broader support for intravascular imaging are necessary in order to fully establish the potential benefits and shortcomings of rota-lithotripsy, before its wider implementation into clinical practice can be achieved.

## Figures and Tables

**Table 1:** The baseline clinical characteristics of the study groups.

Rota-Lithotripsy Atherectomy (RA) N-15	
Age	70.9 ± 9.1
Gender male (ratio)	13 (86.7%)
Unstable angina	2 (13.3%)
NSTEMI	11 (73.4%)
STEMI	2 (13.3%)
Diabetes mellitus	10 (66.6%)
Chronic heart failure	7 (46.7%)
Hypertension	13 (86.6%)
Hyperlipidemia	15 (100%)
Atrial fibrillation	5 (33.3%)
History of PCI	7 (46.7%)
History of MI	8 (53.3%)
History of CABG	2 (13.3%)
COPD	3 (20%)
Chronic kidney diseases	5 (33.3%)
History of stroke	3 (20%)

Abbreviations: NSTEMI—non-ST-elevation myocardial infraction; STEMI—ST-elevation myocardial infraction; PCI—percutaneous coronary intervention; MI—myocardial infraction; CABG—coronary artery bypass grafting; and COPD—chronic obstructive pulmonary diseases.

**Table 2:** The baseline procedural features of both study groups.

Rota-Lithotripsy Atherectomy (RA) N-15	
Syntax I score	21.1 ± 10.9
Syntax II—PCI score	39.0 ± 14.9
Syntax II PCI four-year mortality	22.1 [5.2–23.5]
Syntax II—CABG score	36.3 ± 8.7
Syntax II CABG year mortality	14.1 [7.6–18.3]
Radial access	12 (80.0%)
6F guide catheter	4 (26.7%)
7F or larger guide catheter	11 (73.3%)
Initial unsuccessful predilatation	9 (60.0%)
Rota burr diameter (mm)	1.55 ± 0.15
Rotablation duration time (s)	111.7 ± 61.2
RPM	162,300 ± 4242
IVL diameter (mm)	3.2 ± 0.15
Number of pulses	45.3 ± 19.9
Intravascular guidance	4 (26.7%)
DES diameter (mm)	3.16 ± 0.48
Total DES length (mm)	64 ± 29.7
Postdilatation	11 (73.3%)
Postdilatation balloon diameter (mm)	3.41 ± 3.95
Postdilatation pressure (atm)	19.72 ± 0.55
Acetylsalicylic acid	15 (100%)
Clopidogrel	6 (40%)
Ticagrelor	6 (40%)
Prasugrel	3 (20%)

Abbreviations: PCI—percutaneous coronary intervention; CABG—coronary artery bypass grafting; POT—proximal optimization technique; and bold text—statistically significant value.

**Table 3:** Clinical outcomes of the study groups.

Rota-Lithotripsy Atherectomy (RA) N-15	
**In-hospital period**	
MACE	1 (6.7%)
Death	0 (0%)
Myocardial infarction	1 (6.7%)
Target vessel revascularization	1 (6.7%)
Stent thrombosis	1 (6.7%)
Target lesion failure	1 (6.7%)
Stent restenosis	0 (0%)
Any revascularization	1 (6.7%)
Cerebrovascular episodes	0 (0%)
Major bleeding	2 (13.3%)
**1-month follow-up**	
MACE	1 (6.7%)
Death	0 (0%)
Myocardial infarction	1 (6.7%)
Target vessel revascularization	1 (6.7%)
Stent thrombosis	1 (6.7%)
Target lesion failure	1 (6.7%)
Stent restenosis	0 (0%)
Any revascularization	2 (13.3%)
Cerebrovascular episodes	0 (0%)
Major bleeding	2 (13.3%)
**6-month follow-up**	
MACE	2 (13.3%)
Death	1 (6.7%)
Myocardial infarction	1 (6.7%)
Target vessel revascularization	1 (6.7%)
Stent thrombosis	1 (6.7%)
Target lesion failure	1 (6.7%)
Stent restenosis	0 (0%)
Any revascularization	5 (33.3%)
Cerebrovascular episodes	0 (0%)
Major bleeding	2 (13.3%)

Abbreviations: MACE— major adverse cardiac events.

## Data Availability

All data not presented in the manuscript are available following a written request being sent to the corresponding author. All data will be provided in accordance with local law restrictions.
